# The gradual coevolution of syntactic combinatorics and categorization under the effects of human self-domestication: a proposal

**DOI:** 10.1007/s10339-023-01140-6

**Published:** 2023-06-12

**Authors:** Antonio Benítez-Burraco, Koji Hoshi, Ljiljana Progovac

**Affiliations:** 1 Department of Spanish, Linguistics and Theory of Literature (Linguistics), Faculty of Philology, University of Seville, Seville, Spain; 2 Faculty of Economics, Keio University, Tokyo, Japan; 3 Linguistics Program, Wayne State University, Detroit, USA

**Keywords:** Merge, Cross-modality, Categorization, Aggression, Language evolution, Human self-domestication

## Abstract

The gradual emergence of syntax has been claimed to be engaged in a feedback loop with Human Self-Domestication (HSD), both processes resulting from, and contributing to, enhanced connectivity in selected cortico-striatal networks, which is the mechanism for attenuating reactive aggression, the hallmark of HSD, but also the mechanism of cross-modality, relevant for syntax. Here, we aim to bridge the gap between these brain changes and further changes facilitated by the gradual complexification of grammars. We propose that increased cross-modality would have enabled and supported, more specifically, a feedback loop between categorization abilities relevant for vocabulary building and the gradual emergence of syntactic structure, including Merge. In brief, an enhanced categorization ability not only brings about more distinct categories, but also a critical number of tokens in each category necessary for Merge to take off in a systematic and productive fashion; in turn, the benefits of expressive capabilities brought about by productive Merge encourage more items to be categorized, and more categories to be formed, thus further potentiating categorization abilities, and with it, syntax again. We support our hypothesis with evidence from the domains of language development and animal communication, but also from biology, neuroscience, paleoanthropology, and clinical linguistics.

## Introduction

For many years, the mainstream view in language evolution studies has been that language can (and actually should) be construed as a human-specific cognitive faculty that resulted from biological changes mostly and that emerged along with our species. Languages, on their part, have been conceived of as by-products of that faculty. Certainly, many different languages exist, with linguistic diversity to some extent being triggered and shaped by factors external to language, such as geographical isolation or specific cultural practices. Nonetheless, all languages, either present-day or past, also share many structural properties, to the extent that they can be considered roughly comparable in terms of their basic architecture and their overall complexity. Importantly, these universal features of languages, particularly, in the domain of syntax, have been regarded as depending on our brain architecture only. In brief, once our language-ready brain emerged, modern grammars were supposed to emerge, too. The Chomskyan account of language evolution best exemplifies this view (see Berwick and Chomsky 2016 for a recent summary).

However, ongoing research in different fields related to language, such as comparative and evolutionary neuroscience, language acquisition studies, language typology, sociolinguistics, or paleoanthropology, is calling into question three basic assumptions of such view: (i) that all languages are, and have been, equally complex; (ii) that their structure is mostly insensitive to the environment; and (iii) that human cognition has remained unchanged since our origins.^1^ Accordingly, some key design features of human language may have resulted from social transmission and cultural evolution (Sandler 2005; Tamariz and Kirby 2016). Likewise, it has been proposed that most, if not all, structural features of a language, including syntactic features, can be impacted upon by the physical, and particularly, the social environment in which it is spoken, the latter including the number of speakers, the degree of bilingualism, the tightness or the looseness of the social networks, or the number of adult learners (Wray and Grace 2007; Lupyan and Dale 2010; Gil 2021).

Finally, ample evidence suggests that our brain (and seemingly our cognition as well, including our ability to learn and use languages) has changed over time, with these changes possibly picking up pace after our split from Neanderthals and Denisovans, and extending to the present day, in a process that parallels to some extent the emergence of behavioral modernity (Neubauer et al. 2018). These changes in our cognition and our behavior can indeed be expected to have affected the nature of the languages spoken by our ancestors. But at the same time, we should also expect that the changes in the languages we spoke also impacted on our cognition and behavior, since the habitual encoding and use of specific language features can result in representational and procedural changes (see e.g., Amici et al. 2019, on the impact of word order on working memory).

In summary, increasing evidence suggests that our cognitive architecture (and let’s include our behavior, too) does account for many aspects of the languages we speak, but also that some language features depend on environmental and cultural factors, and in turn affect, more or less permanently, our cognitive architecture and our behavior. These two aspects cannot be detached one from the other and both seem to be engaged in a mutually reinforcing feedback loop. As a consequence, both biology and culture need to be considered on a par if we aim to understand how language evolved and how languages were in the past. While various approaches to language evolution tend to reduce language evolution to one single factor, whether it is culture, or genes, or Merge, or categorization, our framework recognizes the role of various factors, and moreover proposes specific ways in which their interactions worked to yield the phenomenon as complex as human language certainly is.

In this paper, we rely on the self-domestication hypothesis of human evolution (HSD), as introduced in detail in  Human self-domestication and the evolution of language(s), and more specifically on the model of language evolution under the effects of HSD forces, as introduced in more detail in  The mechanism of grammar complexification under HSD. According to this model, HSD was engaged in a feedback loop with the emergence of simple forms of language, significantly contributing to shaping language structure (Progovac and Benítez-Burraco 2019; Benítez-Burraco and Progovac 2020, 2021) and language use (Benítez-Burraco et al. 2021). Of most relevance to our paper, this feedback loop with HSD has been proposed to have potentiated the control of selected subcortical areas by selected cortical areas, this contributing to the inhibition of reactive aggression, but also, simultaneously, to the enhancement of cross-modality, as both aspects are hypothesized to rely on the same cortico-subcortical circuits (Benítez-Burraco and Progovac 2021). Cross-modality can be characterized as the ability to forge connections among core knowledge systems, including sensory modalities, and is especially relevant for metaphorical extension in language (see e.g., Spelke 2000; Spence 2011; Cuskley and Kirby 2013; Shayan et al. 2014). Evidence for the gradual reduction of reactive aggression in humans over the past 80,000 years or so can be found in the fossil record, as discussed in e.g., Cieri et al. (2014), but it can be inferred as well from direct comparisons with extant primates, as discussed in e.g., Herrmann et al. (2011); Hare et al. (2012).

In this paper, we aim to bridge the gap between these neurobiological and cognitive changes resulting both from HSD and the changes resulting in, and promoted by, the gradual complexification of grammars, as they engaged in a feedback loop. In  The feedback loop between categorization and syntactic combinatorics under the effects of HSD, we will specifically propose that increased cross-modality associated with HSD and the concomitant brain modifications described above, would have enabled and supported not merely an increase of grammar complexity, but more specifically, a feedback loop between categorization abilities relevant for vocabulary building and the gradual emergence of syntactic structure, including the core combinatorial operation in natural languages, such as Merge.^2^ In brief, an enhanced categorization ability not only brings about more distinct categories, but also a critical number of tokens in each category necessary for Merge to take off in a systematic and productive fashion; in turn, the benefits of expressive capabilities brought about by productive Merge encourage more items to be categorized, and more categories to be formed, thus further potentiating categorization abilities, as elaborated in more detail in  The feedback loop between Merge and Categorization in the light of HSD.

As will be discussed in  Evidence from animal communication and language acquisition, the need to amass a critical number of words before breaking into syntax (i.e., Merge) is amply demonstrated in language acquisition studies. But it is equally clear that without Merge, the advancements in language or categorization are not possible. Animal communication abilities, including those of trained animals, seem to stop short of this critical number, which, in our analysis, goes hand-in-hand with them not developing productive syntax, or human-like categorization abilities (Evidence from animal communication and language acquisition). Overall, in this paper we build on evidence from diverse fields, including linguistics, ethology, biology, paleoanthropology, language acquisition, sociolinguistics, language typology, neuroscience, and clinical linguistics.

## Human self-domestication and the evolution of language(s)

As noted above, HSD refers to a recent hypothesis about how our species emerged. It claims that the human distinctiveness is, to a large extent, the outcome of an evolutionary process similar to animal domestication. In mammals, domestication is usually triggered by selection for tameness and results, in most cases, in a constellation of distinctive traits that are physical, cognitive, and behavioral by nature. According to some views, this is due to the fact that tameness reduces the input to the neural crest, an embryonic structure giving rise to many different body parts during development (Wilkins et al. 2014, 2021). A reduction in reactive aggression has been considered to be the main ingredient of HSD (Hare 2017; Hare and Woods 2020).

The hypothesis of HSD builds on the existence in humans of many of the traits commonly found in domesticated varieties of mammals, including reduced skulls/brains, at least in recent specimens; childish facial features; reduced body hair; prolonged childhood; increased playing behavior, and particularly, a less aggressive behavior. Among the factors commonly cited in the literature that might have triggered HSD, one finds the rise of community living, the advent of co-parenting, changes in our foraging ecology, the increasingly harsh environments resulting from the Quaternary Glaciation, and/or the colonization of new environments (Pisor and Surbeck 2019; Brooks and Yamamoto 2021; Spikins et al. 2021). All these factors seemingly promoted a selection toward less emotionally reactive partners and toward tolerance for extra-group individuals. In turn, the physical, behavioral, and perhaps even cognitive changes brought about by HSD are claimed to have favored the emergence of many human-specific distinctive features, including our enhanced social cognition, increased cooperation and extended social networks, and ultimately, our advanced technology and sophisticated culture (Hare 2017; see Hare and Woods 2020, for details).

However, it was the finding that in some birds, domestication results in more complex communicative signals (e.g., Okanoya 2017) that paved the way toward claims that HSD could be extremely valuable in capturing key aspects of the evolution of language, specifically, the aspects that are thought to emerge through a cultural mechanism (Thomas and Kirby 2018). Ongoing research by Benítez-Burraco and Progovac (2020, 2021) has crystallized in a detailed account of how HSD might have contributed to the evolution of language (and of languages) in our species, pursuing a model which involves a feedback loop between the two, and which encompasses four distinct, but overlapping stages (as elaborated in  Step-by-step: Linking syntax-related categorization with a gradual model of language evolution under HSD forces).

## The mechanism of grammar complexification under HSD

As noted in the previous sections, one of the key contributors to the complexification of grammars under the effects of HSD was the creation (and the progressive sophistication, as features of HSD increased) of the niche that facilitated this complexification through a cultural mechanism. As also noted, this niche encompasses behavioral changes that augmented the quality and quantity of interactions between individuals, as well as the opportunities for teaching, learning, and practicing through play. However, because animal domestication has proven to impact on cognition, too, one can expect that HSD impacted as well, even if subtly, on our brain structure, and ultimately, on our cognitive abilities, including language processing abilities. In Benítez-Burraco and Progovac (2021), it is hypothesized that the reduction in reactive aggression levels brought about by HSD is accompanied by an increased inhibition by the cortex of the subcortical mechanisms involved in reactive aggression responses, this ultimately entailing an increased connectivity between selected cortical areas and striatal networks.

Importantly, these authors further argue that, because these cortico-subcortical networks are also critically involved in cross-modality (as noted, the ability to transcend the boundaries of core knowledge systems, and to utilize metaphoricity), one consequence of the reduction in reactive aggression levels would have been the potentiation of our cross-modal abilities. In turn, as cross-modal activity is ultimately involved in the creation of metaphorical compounds and in metaphors more generally, which are important for both grammaticalization and Merge, one can expect that language complexity and language processing generally would have improved, too, engaging in a feedback loop with HSD processes, and thus further impacting on the evolution of the cortico-striatal networks. Incidentally, this was proposed to be the reason why altered cross-modality, increased reactive aggression, and problems with language structure and use (including metaphorical, figurative uses of language) cluster together in most cognitive disorders (see Benítez-Burraco and Progovac 2021, for details).

Overall, this model provides a unitary view of different traits that are involved in language evolution, including cross-modal thinking and rule-governed systematicity (important for the evolution of the cognitive hardware of language), and the control of aggression (important also for socialization and the cultural evolution of language). Additionally, it reveals a clear continuity between human language and the cognitive abilities and behaviors exhibited by other species, thus providing a more robust bridge between biological and cultural accounts of language evolution, but also a smoother transition from animal cognition and behavior to human cognitive abilities and behavioral features (including language).

That said, an important gap still exists between the broad neurobiological changes to which HSD contributed, as described above, and the specific changes affecting grammars and vocabularies. In an attempt to narrow this gap even further, this paper adds a new component to this general approach by considering the role of categorization in vocabulary building and in the evolution of Merge, and syntax more generally. Roughly speaking, we will argue that the increased cross-modality brought about by the potentiation of brain connectivity in humans, initially associated with reduced reactive aggression, enabled us to recruit preexisting categorization abilities, as found in non-human animals and other hominins. The constructive feedback loop that ensued between these improved categorization abilities and the emergence of simple syntax, i.e., (proto-)Merge, significantly improved upon both, enhancing both our syntactic combinatorial power and our categorization power, gradually contributing to higher cognition in our species. We now provide a more detailed characterization of our hypothesis, as well as comparative, developmental, and neurobiological evidence supporting such view.

## The feedback loop between categorization and syntactic combinatorics under the effects of HSD

### Categorization

Categorization can be defined as the cognitive process of grouping different entities/events based on perceived similarities among them (see Hoshi 2018, 2019, and references there for many details).^3^ Some authors (e.g., Spinozzi et al. 1999; Conway and Christiansen 2001; Penn et al. 2008; Bouchard 2013, among others) have claimed that only humans can engage in categorization of categories, in addition to categorization of individual objects and events. Nonetheless, ample research (e.g. Roberts and Mazmanian 1988; Vonk and McDonald 2002, 2004, among others) reports that pigeons, monkeys, gorillas, and orangutans are capable of dealing with categorization of categories to some extent (see Mareschal et al. 2010, for an in-depth discussion on categorization in both humans and non-human animals). As a concrete illustration of such advanced categorization in non-human animals, Blumstein (2007) and Rundus et al. (2007) report that California ground squirrels not only distinguish between their predator snakes and other stimuli in the external world, but also differentiate rattlesnakes from gopher snakes, to the extent that they display different defensive responses to the two kinds of predators. Likewise, Hauser (1998) and Hauser and Marler (1993) report that rhesus monkeys can categorize food into high-quality food and low-quality food, as well as create a category of food as a whole, further associating different calls to each category.^4^ Overall, it is reasonable to expect that this ability characterizes many vertebrates, and that it is indispensable for surviving in a world that includes an almost infinite variety of objects and events (see Lenneberg 1967; Bickerton 1990, among others, for this view). That said, while non-human animals, as well as humans, seem to be capable of categorization in this sense, only humans can attach linguistic labels to categories for their identity, as originally pointed out by Lenneberg (1967).^5^ This allows humans to create hundreds or more abstract categories, the vast majority of which are not related to survival. In this sense, humans can be seen as super-sophisticated categorizers.

Let us now illustrate how categorization (henceforth, Cat) works. Imagine that there are three oranges (a, b, and c) in front of you; then, because of their perceptual resemblance, you could put the three oranges into a group under the label “ORANGE” by bringing the cognitive representations [a], [b], and [c] for a, b, and c into a common set A with the label “ORANGE” ({[a], [b], [c]}, where a, b, c ∈ ORANGE). By the same token, you can create other category sets like B = category set of lemons and C = category set of grapefruits, in addition to A = category set of oranges. Then you could put the three different category sets into a super-category set under the label “CITRUS” by bringing the corresponding cognitive representations [A], [B], and [C] into such super-category set with the label “CITRUS.” This sort of Cat targets cognitive representations of category sets rather than those of individual objects (see Hoshi 2018, 2019, for more discussion on Cat). (1) sums up the above-mentioned illustration.

1 $$\begin{gathered} a.\;Cat^{{ORANGE}} \left( {[a],\;[b],\;[c]} \right) = \left\{ {[a],[b],[c]} \right\},where\;a,\;b,\;c \in ORANGE \hfill \\ b.\;Cat^{{CITRUS}} \left( {[A],\;[B],\;[C]} \right)  = \left\{ {[A],\;[B],\;[C]} \right\},where\;A,\;B,\;C \subset CITRUS \hfill \\ \end{gathered}$$

More generally, if *κ* is the variable of a label for Cat and if [*α*] is the cognitive representation of *α* in the brain, where *α* is a variable ranging over objects/events or category sets of objects/events or category sets of category sets (of objects/events), then, the category label *κ* can be taken as a sort of characteristic function that applies to any element indicated by [*α*] that either “satisfies” the label *κ* or not, as defined in (2):^6^^,^^7^

2 $$K([\alpha ])\left\{ \begin{array}{ll} 1&\quad{\rm if}\,[\alpha]\;{\text{satisfies}}\;K\\ 0&\quad{\rm if}\,[\alpha ]\;{\text{does}}\;{\text{not}}\;{\text{satisfy}}\;K \\ \end{array} \right.$$

Hoshi (2018, 2019) argued that recursion can be observed in Cat as well, to the extent that it can be defined as a n-ary recursive set-formation cognitive operation. Accordingly, Cat can always yield one more superset category with an appropriate category label, as formulated in (3):

3 $${\text{cat}}\;\kappa ([\alpha ])\;1,...,[\alpha ]{\text{n}}) = \{ \left. {[\alpha ]{\text{i}}} \right|\kappa ([\alpha ]{\text{i}})\} \;\;\;\;{\text{(1}} \le {\text{i}} \le {\text{n)}}$$

As previously noted, some form of categorization is shared with other animals and, accordingly, can be expected for other hominins, too. Our argument here is that our enhanced HSD reinforced these pre-existing categorization abilities via the potentiation of cross-modality, resulting in expanded vocabularies. In turn, this expanded lexicon would have enabled the complexification of grammar through a semantic bootstrapping mechanism, but also through grammaticalization, i.e., the creation of abstract grammatical categories by Cat through metaphorical extension processes, for which cross-modality is indispensable. Now, more complex grammars were able to select more categories with labels (= concepts) that can combine, thus favoring the creation of new categories by Cat. As a consequence, category creation and syntax complexity can be hypothesized to be engaged in a positive feedback loop, under the effects, we wish to argue, of HSD forces. Importantly, as also noted earlier, HSD has the effect of reducing emotional responses (i.e., reactive aggression) to external stimuli and increasing control of the pre-frontal cortex over the striatal regions. As such, it would have led to less dependence on the emotion-charged sensorimotor immediate experience, as found in other species, enabling the detachment of Cat from the sensorimotor domain, and ultimately the creation of more abstract and more diverse categories, transcending, as noted, the boundaries of the core knowledge systems.^8^ Below we provide a more detailed account of this view (subsection The feedback loop between Merge and Categorization in the light of HSD), as well as diverse types of evidence supporting it: comparative and developmental (subsection Evidence from animal communication and language acquisition) and neurobiological (subsection Evidence from neurobiological considerations).

### The feedback loop between merge and categorization in the light of HSD

We propose the following way that the evolution of syntactic combinatorics (i.e., Merge) was entangled with several other evolutionary developments, specifically categorization. Early in the evolution of language, reduced reactive aggression, associated with HSD, started to enhance cross-modality by increasing cortico-subcortical connectivity (Benítez-Burraco and Progovac 2021). This is so because, as noted, the mechanism for curtailing reactive aggression in humans is enabled by dense connectivity in the cortico-striatal networks, and so is cross-modality (associated with metaphoricity) and syntax more generally (associated with Merge). In this view, there existed a mutually reinforcing feedback loop between the emergence of simple proto-Merge^9^ and the forces leading to the suppression of reactive aggression, both precipitating changes in the same brain networks.

Given that (proto-)Merge combines elements of distinct categories, such as noun-like categories denoting individuals and verb-like categories denoting actions (e.g., *rattle-snake; stink-bug; Eagles fly; Drink water*), the availability of Merge certainly favored categorization and naming of more and more entities/actions into such categories, rendering Merge more and more prolific and productive, as a richer categorization of the world can certainly be expected to be adaptive. A consequence of this would have been, on the one hand, the reinforcement of the previously evolved categorization abilities as observed in other species (Categorization). But, on the other hand, as categorization abilities increased, including an increasing number of categories due to the enhancement of availability of syntactic objects as category labels, another consequence of this would have also been the reinforcement of Merge itself and the further complexification of syntax, resulting from the introduction of abstract grammatical categories (The mechanism of grammar complexification under HSD ) and layers, i.e., hierarchical structure (see below).

For concreteness’ sake, suppose that at the point when our ancestors began using proto-Merge to compose expressions such as *Eagles fly*, they also started wondering what else can fly, and this led to naming and categorizing of other flying individuals, perhaps first those with wings (e.g., *Robins fly; Flies fly*). Furthermore, this strategy would have been extended to flying things without wings (e.g., *Leaves fly; Sand flies*), until, much later, it could even be used for quite radical metaphorical extensions, to express something unobservable, such as *Time flies*, for which cross-modality is especially relevant. And then suppose that our ancestors started wondering what else eagles can do, in addition to flying, perhaps drink, and then enhancing the category of animates who can drink (e.g., *Eagles drink; Lions drink; I drink*). This now expands the category of nouns significantly.

At the same time, our ancestors would have started to wonder what else eagles or lions can do, which encouraged them to give names/category labels to other actions (e.g., *Eagles soar; Eagles fall; Eagles sink*), which now expands the category of verbs, and can further lead to a host of metaphorical extensions, such as *Heart sinks*, or *Heart soars*, for which enhanced cross-modality is again especially useful; and so on and so forth with each noun and each verb. In stark contrast, in the one-word stage there is much less utility in just naming these actions and individuals, and much less, if any, possibility for metaphorical extension, as cross-modality was low or even absent, correlated with the high levels of reactive aggression. The question is how motivated one would be to learn and memorize hundreds, or even thousands of names/category labels for verbs such as *soar*, or *fly*, or *fall*, if they cannot be easily attributed to some entities/individuals, and if they cannot be metaphorically extended, as per the examples above.

It is worth noting that metaphorical extension can only work its wonders when there is a possibility of Merge, that is, syntax. So, in this view, the advent of (proto-)Merge would have enabled the expansion of the vocabulary, as well as the processes of metaphorical extension, which would have in turn contributed to the entrenchment and the agility of Merge, all of this resulting from, but also further enhancing, cross-modality and brain connectivity more generally, in turn fueled by increased HSD.

It is important to emphasize again that metaphorical extension is not something that happens with isolated words in the lexicon; it is really something that happens when words combine in new and unexpected ways. We can only know that the verb *sink* is used metaphorically if it is used with a noun such as *heart*, but not if it is used in isolation. A real breakthrough in the expressive abilities, as well as significant cognitive shift, would have ensued after the relatively static one-word stage of language, with no syntax, gave rise to the much more dynamic stage featuring proto-Merge, both enhanced by cross-modality and contributing to further enhancement of cross-modality.

Furthermore, for the transition from these flat two-slot combinations, formed by proto-Merge, to hierarchical, layered syntax, characterized by full-fledged, recursive Merge, even more abstract syntactic categories needed to emerge, such as transitivity, tense/aspect, and subordination, which build further syntactic layers. These abstract categories such as tense/aspect typically grammaticalize from more concrete lexical (content) categories, e.g., from verbs like *want, go, have, finish* (see e.g., Heine and Kuteva 2007, and references there), involving again metaphorical extension, in turn enabled by enhanced cross-modality. For example, verbs like *want* in different languages grammaticalize into future markers, and verbs like *finish* into aspectual markers for completed actions.

So, to take just one example, grammaticalizing an abstract category of Tense or Aspect (such as *want or finish*), would have yielded creations like *Want eagle fly*, or *Finish eagle fly*, adding another (functional) layer of structure (say Aspect Phrase) to the ancestral two-slot small clause layer, facilitating transition into hierarchical syntax, and a transition from proto-Merge, which only combines two entities, and is not recursive, to Merge, which can apply and reapply, and which eventually can allow Move. Move crucially depends on there being hierarchical structure, given that Move is conceived in this framework to target a hierarchically higher, c-commanding position.^10^

So, in this analysis, Merge and Cat need each other, and are complementary to each other. They are not only partly enabled by enhanced brain connectivity and HSD, but they also in turn contribute to these neurobiological forces. As noted, given our approach, the same brain circuits that are involved in suppression of aggression also support the processing of syntax, as well as cross-modality, associated with metaphoricity, hence the strengthened feedback loop between Merge and Cat under HSD. As pointed out in  The mechanism of grammar complexification under HSD , this is why these three dimensions (metaphoricity, reactive aggression and syntax) also tend to be impaired simultaneously in cognitive disorders that affect language (Benítez-Burraco and Progovac 2021).

Our focus in this paper is on the feedback loop between syntactic combinatorics (Merge) and enhanced categorization abilities, that is, on understanding how they co-evolved gradually by reinforcing each other. As such, our proposal is well-positioned to explain why there exist no communication systems with thousands of vocabulary items, but no syntax, including among natural languages and the stages in child language acquisition, but also in animal communication systems. Accordingly, there is just no culture, and no individuals, who command a vocabulary of say 10,000 words, or even 1000 words, but who do not employ syntax. It would certainly be possible to imagine a language acquisition scenario where children first learn thousands of words (reflecting their abilities to recognize a wide variety of concepts) before starting to combine them. But this just does not happen, as elaborated in the following section.

### Evidence from animal communication and language acquisition

When it comes to trained animals, including primates, they typically do not acquire more than 100–200 or so word-like items, whereas human languages operate with tens of thousands of words. For the primates, one can conclude that this limit on their vocabulary size reflects the abilities of communication systems without syntax, rather than some cognitive limitation in how many concepts they can grasp. It has been reported that other primates are in principle capable of very simple two-word combinations, such as *hide peanut* and *hide Kanzi* (see e.g., Greenfield and Savage-Rumbaugh 1990: 161; regarding bonobo Kanzi). As reported in Patterson and Gordon (1993), the gorilla Koko was capable of producing novel compounds, even playful ones. It has also been reported that Washoe, a chimpanzee who learned how to use signs of American Sign Language, combined the signs for *water* and *bird* to describe a duck (Gardner et al. 1989).

These sporadic attempts at combining signs can be seen as resembling the first attempts by our ancestors (as well as young children) at proto-Merge, before they broke into productive Merge-cum-Cat. In this sense, we recognize continuity with other species both when it comes to Merge and categorization abilities ( Categorization): they both have perhaps clumsy but extremely useful precursors in other species. That said, Merge serves to combine distinct, disparate categories, two at a time, such as noun-like and verb-like elements, i.e., argument-like and predicate-like elements. In order for Merge to operate and to become productive, such categories need to be previously established, and perhaps the critical number is about 100 or more spread across these distinct categories. Once Merge becomes productive, it proves to be highly beneficial for expressive possibilities (see previous section for specific examples). This feedback loop can be hypothesized to have operated throughout human evolution, resulting in more and more categories to be combined by Merge, in more and more instances of Merge (more hierarchy) in sentences, and in more and more concepts to be categorized and named. As noted, this analysis is supported by the processes attested in language acquisition.

With regards to child language acquisition, empirical studies have found a strong association between lexical and grammatical acquisition, crucially also at the point when syntax just begins to emerge. The onset of grammar seems to be directly related to the size of the lexicon (Thordardottir et al. 2002), suggesting that children need to acquire the critical mass of words first before starting to combine them (Bates and Goodman 1997; Thordardottir et al. 2002), and this number is typically reported to be at around 100–200. For example, Stolt et al. (2009) studied the emergence of grammar in relation to lexical growth in Finnish children (*N* = 181) at the age of 2;0. The onset of grammar occurred in close association with vocabulary growth, with the strongest growth of e.g., case form types occurring when the nominal lexicon size was roughly between 50 and 250 words, confirming again the relevance of this approximate number.^11^ Moreover, because their study targeted children at a fixed age of 2;0, the conclusion is that this correlation is not with age, but rather specifically with the size of the vocabulary itself.^12^

This kind of strong correlation between the size of the lexicon and the acquisition of grammar has been found to exist across various cultures/languages: in English children (Anisfeld et al. 1998; Bates et al. 1988; Bates and Goodman 1997; Dionne et al. 2003); in Italian children (Caselli et al. 1999); in Hebrew children (Maital et al. 2000); in Icelandic children (Thordardottir et al. 2002); in German children (Szagun et al. 2006); and in Finnish children (Lyytinen and Lyytinen 2004).

What is more, many researchers of language acquisition have converged on the view that vocabulary acquisition and the acquisition of syntax are mutually reinforcing and mutually constraining (Bates and Gooodman^1997^; Thordardottir et al. 2002; Goodman and Bates 2013). This is consistent with our proposal of the evolutionary feedback loop in which the growth of the lexicon through categorization, and the growth of grammar are entangled, and reinforce each other. In language acquisition literature, the mechanism behind this association is typically referred to as *bootstrapping* (e.g., Pinker and MacWhinney 1987; Abend et al. 2017).

On the one hand, children’s vocabulary growth is foundational for acquiring grammar (*semantic bootstrapping*). On the other hand, children also rely on the syntactic context of sentences to acquire (the meanings of) new words (*syntactic bootstrapping;* Gleitman 1990; Gleitman and Gillette 1999; Fisher et al. 2010). It seems that there is greater reliance on the former mechanism (semantic bootstrapping) at the early stages of acquisition and more reliance on the latter mechanism (syntactic bootstrapping) at the later stages. Caglar-Ryend and colleagues (2019) found that the shift in favor of syntactic bootstrapping occurs somewhere at the age of 3–3;5 years old, with syntax continuing to play a leading role thereafter.^13^ Wagley and Booth (2021) provide robust evidence for syntactic bootstrapping in children ages 6–7;5 years old.

Consistent with our proposal, many researchers relate the association between vocabulary size and syntax (semantic bootstrapping) to the need to form classes and categories first, before starting to develop syntax. In vocabulary acquisition, it is usually observed that children first acquire names (noun-like categories), then predicate terms (verb-like categories), as well as some small grammatical items, as noted by Bates and colleagues (1994). According to their view, children cannot acquire relational terms before they have acquired enough words for the things to which predicate words relate. In this sense, semantic bootstrapping offers a way for a child to get hold of word classes and of how different types of words are used in grammatical structures (Clark 2003). This means that children need to form robust enough categories before they can start juxtaposing them and combining them in a productive fashion, enabling the functioning of Merge.

In sum, both animal communication studies and language acquisition studies are consistent with our proposal that categorization, related to vocabulary building, and syntax, including Merge, rely on each other and reinforce each other, in the sense that a critical mass of words is needed before Merge and syntax can take off, as well as that Merge and syntax encourage further categorization and vocabulary expansion. Animal communication abilities, including those of trained animals, seem to stop short of this critical number, which, in our analysis, correlates with them not developing productive syntax, or human-like categorization abilities. Nonetheless, these animal capabilities are important, in the sense that they provided necessary precursors that our ancestors were able to use to bootstrap themselves to language.

### Evidence from neurobiological considerations

As noted above, cortico-striatal networks are essential not only for the curtailing of reactive aggression in humans (associated with HSD), but also, simultaneously, for the enhancement of cross-modality (associated with metaphoricity), and for syntax more generally (including Merge) (see Benítez-Burraco and Progovac 2021, for details). The neurobiology of Cat includes cortical regions (the pre-frontal cortex, the visual cortex, the anterior cingulate, and the medial temporal lobe) and subcortical regions (the basal ganglia and the hippocampus), depending on various categorization tasks (see e.g., Ashby and Ell 2001; Ashby and Ennis 2006; and Ashby and Crossley 2010). In particular, among the array of brain areas, the basal ganglia (particularly, the striatum) are known to be especially important for Cat in animals including humans; more specifically, the pre-frontal-basal ganglia network is crucial for language-related Cat, where the category labels can be verbally expressed (see the works cited above and references cited therein). The reader is also referred to Seger (2008) and Villagrasa (2018), inter alia, for the point that the interaction between the pre-frontal cortex and the basal ganglia is deeply involved in Cat in humans.

Interestingly, with regards to cross-modality, and particularly to visual and auditory cross-modal information integration in humans, Smith et al. (2014: 13) note that “in the striatal categorization system (which may be the older vertebrate behavioral-categorization system), cognitive evolution may have emphasized nonmodal signals for adaptive behavior within which the dimensional components of the signal are submerged,” suggesting that “nonmodal”, i.e., liberal/neutral cross-modal nature of the striatum in the basal ganglia is at work for Cat in humans. In order to accept such diverse concepts from multiple domains in a cross-modal fashion, the system of Cat has to be “liberal” or “neutral” so that it will not be biased toward recruitment of concepts from only specific domains such as colors and numbers for category labels.

Likewise, it has been argued that the basal ganglia subserve the function of implementing the hierarchical syntactic structuring (Balari and Lorenzo 2013; Balari et al. 2013). In particular, it is well-known that there exists a crucial cortico-subcortical ‘syntax’ pathway linking Broca’s area and the striatum in the basal ganglia (see e.g., Gibson 1996; Lieberman 2000, 2009; Vargha-Khadem et al. 2005; Ullman 2006; Teichmann et al. 2015; Ardila et al. 2016a,b; Progovac et al. 2018; Hoshi 2019; Murphy 2021; Murphy et al. 2022; Benítez-Burraco et al., forthcoming). In this respect, Teichmann et al. (2015) identified the BA45-left caudate head pathway in combination with the dorsal arcuate-BA44 pathway as responsible for phrasal level syntactic processing (see also references cited therein).^14^

Overall, the evidence reviewed is consistent with our view here that the syntactic hierarchical combinatorics of Merge co-evolved with the growth of Cat and vocabulary, supported by increased cross-modality, ultimately resulting from enhanced connections between selected cortical regions and selected subcortical areas. Murphy and colleagues (2022) provide a detailed discussion of the shared neurobiological substrate of Merge and Cat and how the involved thalamo-cortico-basal ganglia networks are functionally connected through cross-frequency coupling.

As noted, our hypothesis here is that the enhanced connections between selected cortical regions and selected subcortical areas that increased cross-modality and potentiated Merge and Cat, resulted, at least initially, from the reduction in reactive aggression levels as HSD increased. There are several reasons to believe that cross-modality, Cat, Merge and aggression rely on a common neuronal substrate, hence the feedback loop. First, aggression is mediated by hardwired brain circuitries that specialize in processing certain sensory inputs to trigger stereotyped motor outputs (Lischinsky and Lin 2020), in parallel with sensorimotor categorization. Second, although aggression depends on an evolutionarily conserved ‘core aggression circuit,’ composed of four subcortical regions outside the Merge/Cat circuit, there is also a circuit for aggression-mediated associative learning which involves dopamine signaling in striatal circuits: in brief, the activation of the striatum results in learned aggressive actions. Third, the forebrain (particularly, the pre-frontal cortex) acts as a ‘top-down’ controller of these mechanisms, mostly via serotonin innervation.

For controlling aggression, the pre-frontal cortex interacts mostly with the hypothalamus (part of the ‘core aggression circuit’), but also with the striatum (part of the ‘learned-aggression circuit) (Cupaioli et al. 2021). Here, the interaction between the pre-frontal cortex and the dorsal striatum accounts for the “cognitive” control of aggressive responses, but increased control of striatal regions by the cortex is also expected to result in increased cross-modality, Merge, and Cat.

Finally, as expected in our line of analysis, diseases resulting from striatal dysfunction feature increased reactive aggression, problems with structural language, and problems with figurative language, as in Huntington’s disease or Parkinson’s disease (Savage 1997; Rosenblatt and Leroi 2000; Zgaljardic et al. 2003). While these neurobiological considerations do not constitute ultimate proof of the evolutionary scenario that we have proposed, they are nonetheless consistent with this scenario, which thus remains a viable hypothesis of human evolution. Not only that, but our hypothesis sheds novel light on why these phenomena that characterize recent human evolution cluster together in e.g., cognitive disorders (Benítez-Burraco and Progovac 2021).

## Step-by-step: linking syntax-related categorization with a gradual model of language evolution under HSD forces

For the reasons provided in  Categorization, it is quite reasonable to assume that the syntax-related categorization in the evolution of human languages initially relied on sensorimotor systems, such as perceptual categorization, as available in non-human animals (Hurford 2007, and references there). Its complexity then gradually increased in the evolution of our species, independently of the perceptual categorization, but entangled with other processes relevant to language evolution. In this section we focus on the interaction between the evolution of syntax-related categorization and a gradual model of evolution of languages under the influence of HSD forces, as proposed by Benítez-Burraco and Progovac (2020, 2021), ultimately providing a more detailed account of the gradual coevolution of syntactic combinatorics and categorization.

The model encompasses four stages, although these stages should be better viewed as sections within a continuum, with the transition from one grammar type to another being progressive, as HSD also increased (and later decreased) in a gradual fashion. Even in present-day languages, the complexity of the linguistic structures we use for communicating differs vastly according to the settings in which language is used, from single words and expletives, as found in verbal aggression/contest, to multilayered sentences, as found in a novel. Features of HSD also seem to present variably in different human groups (e.g., Gleeson and Kushnick 2018).

Stage 1 in this model starts with the emergence of our species, roughly 300 kya, although a deeper timeline is not excluded by the model.^15^ Because reactive aggression was still high at that point, communication through language could not have involved patient and cooperative turn-taking, using long utterances, but just single-word commands, threats, and exclamations, mostly aimed to convey emotion, such as (4). If this was the case, then Stage 1 Cat could have assigned a category label such as [x RUN] for the first example in (4), where x stands for some unexpressed entity that was running in the relevant context, as in (5) (in what follows, [] is used for depicting any category label just for expository purposes).

4 $${\text{Run! Go! Move! Up! Down! Look! Bite! Fire! Snake! Eagle!}}$$

5


It can be surmised that those “one-word utterances” in the proto-language Stage 1 were strongly attached to the sensorimotor system. Accordingly, here, there is only a simple Cat with no cross-modal embedding of subcategories.

At some point, features of HSD started to increase, laying the foundations of the cultural transmission process that fuels the sophistication of linguistic structures. At this early period of our history, this was mainly facilitated by the principal physiological and behavioral outcome of domestication: reduced reactive aggression. Slightly lower levels of reactive aggression were surely crucial for establishing stronger in-group networks, involving more diverse, frequent, and prolonged contacts between members. In turn, this resulted in more time and opportunities for learning and teaching, and ultimately, for iterated learning and cultural transmission. A consequence was that both language structure and language use started to complexify.

This is the Stage 2 in the model. Because, as noted, HSD can be conceived of as a gradual, incremental process, it is difficult to provide exact dates for the starting point and the duration of this Stage 2. Still, if environmental changes had a significant impact on HSD, as suggested, one could hypothesize that Stage 2 spanned the time period between roughly 200 kya and 110 kya, corresponding to the long Riss Glaciation and the subsequent Riss-Würm Interglacial period. For this Stage 2, proto-words can be hypothesized to have started to be combined in a pair-wise fashion (primarily combining proto-nouns with proto-verbs), basically leading to rudimentary two-slot grammars that would have been used for describing and characterizing objects, persons, and events, as in (6).

6 $$\begin{gathered} {\text{a)}}\;\;\;{\text{Look snake! Eat fruit! Kill snake! Eagle fly!}} \hfill \\ {\text{b)}}\;\;\;{\text{rattle - snake; stink - bug; scatter - brain; cry - baby; kill - joy; spin - butt (fidget)}} \hfill \\ \end{gathered}$$

Specifically, it is hypothesized that these early grammars were particularly useful for creating colorful derogatory expressions (Progovac 2015). This would have provided a resource for replacing physical aggression by verbal aggression, which is less costly, reinforcing the trend toward a reduction in the levels of reactive aggression (see Progovac and Benítez-Burraco 2019, for details). Overall, this would have resulted in an accelerated feedback loop between early forms and uses of grammar and HSD, with reduced reactive aggression contributing to the increased sophistication of grammars and with increasingly complex linguistic structures contributing, in turn, to further reduction of reactive aggression. As discussed in detail, the ultimate reason for this is the existence of a common underlying mechanism, the enhancement of cortico-striatal brain networks, supporting these two core dimensions of language (and of language evolution), namely, inhibition of reactive aggression and the emergence of cross-modality (including our ability to Merge linguistic items). (7) illustrates Cat under the label [{EAGLE, FLY}] for the last example in (6a).

7 $$\left[ {\left\{ {{\text{EAGLE}},{\text{FLY}}} \right\}} \right]$$

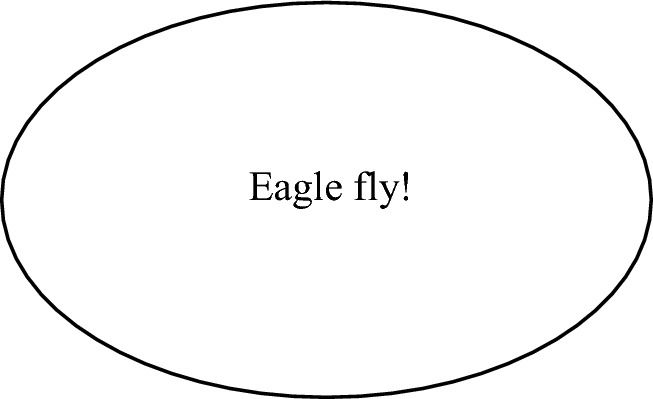


Note that the category label indicated by [{EAGLE, FLY}] shows that the noun-like element EAGLE and the verb-like element FLY are combined by proto-Merge in a non-hierarchical, flat way, i.e., in a small clause (SC) structure, with [{EAGLE, FLY}] being utilized for the category label at hand.

At some point around 50 kya, HSD probably reached its peak, as suggested by paleoanthropological evidence (e.g., Cieri et al. 2014). This is a moment when sophisticated cultural and technological practices flourished in many parts of the world, including in Europe. This can also be regarded as the peak of our Stage 3, whose onset can be placed roughly at 110 kya, when the Würm Glaciation started and when the first evidence of behavioral modernity appeared in different places, too (Mcbrearty and Brooks 2000). Accordingly, the levels of reactive aggression continued to decline during this stage, at an even higher rate than during Stage 2, arguably due to both an increase in language sophistication and to the frequency and diversity of contacts among people, at least among kin, this resulting in enhanced teaching and learning.

Specifically, given that increased HSD results in an extended juvenile period, Stage 3 provided an extended learning/socializing period for children, with more opportunities to acquire language features and abilities through play and other types of interaction. Overall, these changes would have correlated with the emergence of more sophisticated forms of grammar, including the first hierarchical grammars expressing e.g., transitivity. For this stage, the intense feedback loop between HSD forces and early grammars, both contributing to the gradual increase in brain connectivity, would have resulted in increased cross-modality, in turn contributing to further differentiation of Cat in our ancestors by dividing an event-denoting category into two sub-categories of a prominent event participant (= subject) and of a sub-event, where the sub-event was differentiated into two sub-categories of an action and its related sub-event participant (= object). This kind of complex Cat is not possible without enhancement and employment of cross-modal Cat, given the rather abstract relation between the object-denoting category *cats* and the sub-event-denoting category *roll balls*, as illustrated in (8) for the transitive example *Cats rolls balls*:

8 $$\left[ {\{ {\text{CATS}},\{ {\text{v}},\{ {\text{ROLL}},{\text{BALLS}}\} \} \} } \right]$$

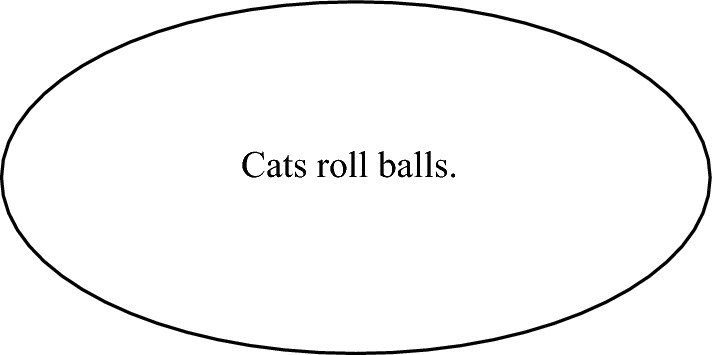


The functional element *v* of the little verb phrase (*v*P) involved in transitivity is the abstract linguistic construct for grammaticalizing the relation between the prominent participant-denoting category and the sub-event-denoting category. Thanks to the further differentiation mentioned above, Stage 3 clearly has hierarchical syntax. In our model, the further cross-modality progresses, the more diversified category labels can become, combining disparate concepts from across various modules, or core knowledge systems in the sense of Spelke (2000) and Spelke and Kinzler (2007) (see also Boeckx 2010).

Finally, as population sizes increased, and seemingly also because of other changes (such as changes in human foraging ecology or climatic changes, particularly, the end of the Last Glaciation), inter-group contacts spread, and extensive social networks emerged, relevant for trading and mating. As a consequence, the necessity of exchanging de-contextualized meanings and know-hows with strangers also increased. According to our view, this correlated with the gradual emergence of the features associated with so-called *exoteric* languages (in the sense of Wray and Grace 2007). These languages are characterized as exhibiting expanded vocabularies, greater semantic compositionality and transparency, as well as increased syntactic complexity, featuring more grammaticalized distinctions and layers, including TP (tense phrase) and CP (complementizer phrase) layers, and with it also greater reliance on embedding and recursion, as in (9):

9 $${\text{[TP I wonder [CP if [TP John will admit [CP that [TP he ate all the bagels]]]]]}}$$

These higher functional projections such as TP and CP are even more abstract, and require further grammaticalization via metaphorical extension, and thus heavy reliance on cross-modality, as well as on complex, abstract labeling in Cat. Importantly, with the introduction of CP and TP in syntactic structures, there was less reliance on the immediate context of the utterance, and less dependence on the emotion-charged sensorimotor immediate experience, thus facilitating inter-group communication, as well as communication across distances. This is Stage 4 in Benítez-Burraco and Progovac’s model, whose starting point can be tentatively situated at 10 kya, with the advent of the Holocene period and the transition from the Paleolithic to the Neolithic.

In this model, the advent of exoteric-type languages can be linked to the advent of new forms of aggression, specifically, proactive (that is, premeditated) aggression, that became wide-spread during this period, and which is rare in other primates, especially in those claimed to have gone through a self-domestication process, like bonobos (Wrangham 2018). Increased proactive aggression in humans has been argued to result from group selection in favor of risk-prone altruism (Choi and Bowles, 2007). Our contention here is that this trend toward more proactive aggressive behaviors might have been facilitated by the advent of exoteric-type languages, which provide precise linguistic tools necessary for intricate planning and knowledge sharing with strangers, thus supporting, among other features, the emergence of cultural institutions around war in complex societies.

## Conclusions

Our narrow focus in this paper is on the feedback loop between syntactic combinatorics (Merge) and enhanced categorization abilities, that is, on understanding how they co-evolved gradually by reinforcing each other, under the influence of HSD. Here we build on a previous proposal that the gradual emergence of syntax in language evolution was engaged in a feedback loop with the effects of HSD, with both processes contributing to enhanced connectivity in the cortico-striatal networks, which is the mechanism for suppressing reactive physical aggression, the hallmark of HSD, but also the mechanism of cross-modality, relevant for metaphoricity, as well as for syntax. In this paper, we highlight the linguistic aspects of this equation, specifically the relation between the evolution of syntax and the evolution of categorization, and propose that increased cross-modality associated with the brain modifications described above would have enabled and supported a feedback loop between categorization abilities relevant for vocabulary building and the gradual emergence of syntactic structure, including the core combinatorial operation in natural languages, such as Merge.

In our proposal, an enhanced categorization ability not only brings about more distinct categories, but also a critical number of tokens in each category necessary for Merge to take off in a systematic and productive fashion; in turn, the benefits of expressive capabilities brought about by productive Merge encourage more items to be categorized, and more categories to be formed, thus further potentiating categorization abilities, and with it, syntax again. The need to amass a critical number of words before breaking into syntax is clearly demonstrated in language acquisition studies. It is also clear that without breaking into syntax there is no advancement in language or categorization abilities. Animal communication abilities, including those of trained animals, seem to stop short of this critical number, which, in our analysis, correlates with them not developing productive syntax, or human-like categorization abilities. In this respect, our proposal is well-positioned to explain why there exist no communication systems with thousands of words, but no syntax, whether in (adult) natural languages, in language acquisition, or in animal systems. At the same time, our gradual approach recognizes the continuity with combinatorial and categorization abilities found in other species.

In addition to the evidence from linguistics, language acquisition, comparative studies, and neurobiological considerations, our proposal is also consistent with the other types of evidence from biology, neuroscience, paleoanthropology, and clinical linguistics. As previously proposed, cortico-striatal networks are essential not only for curtailing reactive aggression in humans (associated with HSD), but also, simultaneously, for the enhancement of cross-modality (associated with metaphoricity), and for syntax more generally. Given our proposal in this paper regarding Cat, it is not a coincidence that neurobiology of Cat implicates overlapping brain regions, and in particular the pre-frontal-basal ganglia network. It is also expected under this general approach that the three dimensions (metaphoricity, reactive aggression and syntax) will be impaired simultaneously in cognitive disorders that affect language, and this is indeed observed in most, if not all, disorders(Benítez-Burraco and Progovac 2021).

More generally speaking, our approach is gradual, as it relies on continuity with other species when it comes to e.g., categorization, combinatorics, and management of aggression and emotions. It is also an approach that gives an active role to multiple players, which are engaged in highly interactive and dynamic feedback loops, including the feedback loop between the biological and behavioral forces associated with HSD, on the one hand, and, on the other hand, the gradual emergence and complexification of various aspects of language structure through a cultural mechanism mostly, including categorization, syntactic combinatorics, and vocabulary building.

## Data Availability

The materials used in the paper are presented as a list of bibliographical references at the end of the paper.

## Footnotes

1 In this respect, Berwick and Chomsky (2016) state that an infant from a Stone Age tribe in the Amazon would, “if brought to [today’s] Boston, be indistinguishable in linguistic and other cognitive functions” from the rest of the children (p. 54).

2 For concreteness, we are using the term *Merge* in this paper, familiar from the Minimalist Program framework for syntax (e.g., Chomsky 1995), but in fact any other term that refers to syntactic combinations of linguistic constituents can be used instead. Our characterization of Merge will become clearer in the discussion below, where we contrast it with what we see as a precursor to Merge: proto-Merge.

3 Strictly speaking, the target elements of categorization as a cognitive operation are cognitive representations in the brain corresponding to entities/events. Pending the explicit formal characterization of categorization below, we informally treat the targets of categorization as if they were entities/events per se here.

4 The detachment from the influence of the immediate sensorimotor experience would have opened up the pathway toward formation of a variety of abstract atomic concepts to be employed as input to syntactic hierarchical combinatorics (see Fujita and Fujita 2021; see also the notion of Offline Brain Systems in Bouchard 2013, 2021). Interestingly enough, such an escape from the perception dependence may have played a vital role not only in the evolution of human language syntax but also of human language lexicon by bringing “the seeds of an ability to create abstract mental spaces, populated by abstract objects, such as virtues, numbers, nationalities, religions, and grammatical rules” (Hurford 2007: 41).

5 Without delving into the hard problem of defining the general notion of concepts in animals, including humans, let alone the relationship between concepts and words, it seems safe to say that category labels consist of relevant information formed at the sensory and the motor areas, with more abstract information fashioned at the pre-frontal area added on top, depending on species (see Bickerton 1990; Hurford 2007; Bouchard 2013, inter alia).

6 See Murphy (2010) for some relevant discussion on categories and concepts; see also Carey (2009) for a comprehensive, in-depth discussion on the origin of concepts, and Bickerton (1990) for some discussion on differences in nature between human concepts and those of non-human animals.

7 It is important to point out that in the fields of psychology and cognitive linguistics, the criteria for membership determination/identification for categories based on extraction of similarities among category set members are flexible, nuanced and complicated (see Rosch 1973; Lakoff 1987; and Taylor 2003, inter alia). See also Lenneberg (1967, Chapter 8) for some discussion on the characteristics of the classification criteria for categorization.

8 But see an interesting proposal in e.g., Gallese and Lakoff (2005), according to which the sensorimotor system underlies both concrete and abstract concepts.

9 Proto-Merge can be characterized as the earliest, perhaps sporadic attempts at syntax, i.e., as the earliest attempts to combine two items into a single meaningful syntactic unit. Arguably, this kind of Merge was binary in this sense, as is today’s modern Merge, but it did not initially involve hierarchy, and thus did not facilitate either recursion or Move (see e.g., Progovac 2015, for a syntactic reconstruction of proto-Merge and for specific examples of proxies/ “fossils” of the products of proto-Merge). While this ancestral proto-Merge may have lacked many features of modern syntax, such as embedding, tense, transitivity, it was especially good at creating new concepts and ratcheting up categorization abilities, as explicated in the text.

10 Notice also that modern Merge not only creates layers of hierarchy, but also yields headed, endocentric constructions, in contrast to what has been argued for proto-Merge (e.g., Progovac 2015), whose best approximations seem to lack headedness, including the so-called exoteric (non-headed) compounds (*turn-coat; kill-joy*).

11 Interestingly, as noted in Progovac (2016) in the context of the evolution of the lexicon, the Swadesh lists of words also range from 100 to about 200 (https://en.wikipedia.org/wiki/Swadesh_list). Words from Swadesh lists are used by historical linguists to track phonological changes given that they tend to be stable and widespread across cultures.

12 One interesting study found that 18 month-old infants’ comprehension is sensitive to non-local syntactic dependencies involving *wh*-words (Perkins and Lidz 2021), although not infants at a younger age. Children seem to learn vocabulary items and syntactic dependencies gradually, and the acquisition of some may come before the acquisition of others, an interesting topic for further investigation.

13 Consistent with these considerations, neurobiological models of language development posit that comprehension processes in younger children rely on early developing temporal lobe structures, enabling direct mapping of sound to meaning, favoring semantic bootstrapping (e.g., Hickok and Poeppel 2004, 2007; Skeide and Friederici 2016). As the frontal lobe matures in older children, comprehension increasingly relies on the inferior frontal gyrus (IFG) involved in syntactic computations, thus enabling syntactic bootstrapping (e.g., Hahne et al. 2004; Skeide and Friederici 2016).

14 In this connection, see also Okanoya (2017) for discussion on the critical relation between the basal ganglia and complexification of songs of the Bengalese finch from the perspective of the domestication syndrome.

15 In our view, the most likely scenario is that Neanderthals and Denisovans shared Stage 2 with humans, but that Stages 3 and 4 are uniquely human (see e.g., Progovac 2016, for the arguments coming from profound variation across languages when it comes to the expression of the hierarchical layers of syntax, especially transitivity). On the other hand, according to Dediu and Levinson (2013), Neanderthals and Denisovans may have operated with fully-fledged hierarchical language, whether it came from the common ancestor with humans, or whether they evolved their own version of Stage 3 and beyond. We leave this question for future research to resolve.

## Declarations

### Publication history

A previous version of this manuscript has been posted as a preprint to PsyArXiv: https://psyarxiv.com/8ns5w.
